# HEV replication is promoted by blocking the NF-κB signaling pathway through inhibiting FLNa expression

**DOI:** 10.1128/jvi.01977-25

**Published:** 2026-01-30

**Authors:** Yueping Xia, Shuangfeng Chen, Qiangqiang He, Chao Cong, Feiyang Long, Yuan Wang, Huichan Liu, Mengsi Hu, Xiaoxia Hu, Yujie Shen, Liangheng Xu, Yunlong Li, Wenhai Yu, Daqiao Wei, Chuanmao Zhang, Fen Huang

**Affiliations:** 1 Life Science and Technology & Medical Faculty, Kunming University of Science and Technology 47910, Kunming, People's Republic of China; 2 Institute of Medical Biology, Chinese Academy of Medical Sciences and Peking Union Medical College 12501, Kunming, People's Republic of China; Wake Forest University School of Medicine, Winston-Salem, North Carolina, USA

**Keywords:** HEV, FLNa, cytoskeleton, NF-κB signaling pathway, apoptosis

## Abstract

Hepatitis E virus (HEV) is the most common cause of acute viral hepatitis worldwide. Filamin A (FLNa), a cytoskeletal protein, is involved in cytoskeleton remodeling to construct a barrier to infection and participates in virus entry and release. However, how HEV enters host cells and how it is sensed by pattern recognition receptors are largely unexplored. In this study, the role of FLNa during HEV infection was evaluated in patients with HEV infection, animal models, and cell cultures. Notably, HEV interacted with FLNa at the early stage of infection and remarkably inhibited the expression of FLNa *in vivo* and *in vitro*. Its knockdown inhibited the proteolytic degradation of IκB, thus blocking the nuclear translocation of NF-κB. As a result, robust viral replication occurred, and numerous virions were released. Furthermore, the inhibition of FLNa suppressed ubiquitination-mediated degradation to aggravate apoptosis and inflammatory responses. Results indicated that FLNa plays a critical role in the remodeling of the cytoskeletal network during HEV infection. This role may be responsible for the easy entry and escape of HEV from sensing by innate immunity.

**IMPORTANCE:**

Hepatitis E virus (HEV) is the most common pathogen of acute viral hepatitis. The mechanisms by which HEV enters host cells and is sensed by pattern recognition receptors are largely unexplored. In the present study, filamin A (FLNa), a cytoskeletal protein, was significantly inhibited in patients with HEV infection, animal models, and cell cultures. The knockdown of FLNa facilitates viral replication by blocking the nuclear translocation of NF-κB while inhibiting ubiquitination-mediated degradation to aggravate apoptosis and inflammatory responses. This work demonstrates that FLNa plays a critical role in the remodeling of the cytoskeletal network during HEV infection. Such remodeling may be responsible for the entry and escape of HEV from sensing by innate immunity.

## INTRODUCTION

Hepatitis E virus (HEV, genus *Paslahepevirus*) is the main pathogen of acute viral hepatitis, causing approximately 20 million infections every year worldwide and an estimated 3.3 million symptomatic cases of hepatitis E (1). Although HEV infection usually results in self-limiting diseases, it leads to a high mortality rate of approximately 15%–20% in pregnant women with acute fulminant hepatitis (acute liver failure) (2, 3). Chronic HEV infections have been reported in patients with immunodeficiency, such as recipients of organ transplants, individuals with HIV infection, and patients undergoing chemotherapy.

Although HEV is responsible for self-limiting acute hepatitis in humans, HEV infection may become chronic in immunocompromised individuals (4). Its genome is approximately 7.2 kb in length and consists of three open reading frames (ORFs). Among these ORFs (1–3), ORF1 encodes seven domains, namely the methyltransferase domain (Met), Y-domain (Y), X-domain (X), papain-like cysteine protease domain (PCP), hypervariable region (HPR), RNA helicase domain (Hel), and RNA-dependent RNA polymerase domain (RdRp) (5, 6). HEV helicase uses ATP to unravel the RNA double strand (1). Mutations in amino acid residues in Hel reduce viral replication (7). ORF2 encodes capsid proteins, and ORF3 encodes phosphorylated proteins and is associated with the assembly and release of virion particles (8).

Although HEV has been identified for more than 30 years, its interactions with its host, especially its entry and transmission with the cytoskeleton, remain largely unexplored. Viruses must circumvent the physical barrier formed by the actin cytoskeleton and its network of associated proteins to gain entry into cells for productive infection (9). The actin cytoskeleton and its interacting proteins associate with the cell membrane and constitute a barrier to infection. The disruption of the actin cytoskeleton allows viruses to enter cells and induces innate immune responses to clear infections. The molecular mechanisms that link virus-induced physical perturbations to host defense pathways remain unclear (9).

Virus entry is closely related to the dynamic organization of cytoskeletal proteins (10). For example, a study on HEV-like particles revealed that dynamin-2, clausin, membrane cholesterol, and actin are required for the entry of HEV (10). However, the role of filamin A (FLNa), a multifunctional actin-binding protein that binds with actin filaments to form a stable cytoskeleton and participates in viral infection (11–13), remains unknown. FLNa cross-links actin filaments into networks via its N-terminal actin-binding domain (13). It participates in cytoskeleton remodeling to effect changes in cell shape and migration. It interacts with integrins, transmembrane receptor complexes, and second messengers. It modulates internalization and directs intracellular trafficking (14, 15). FLNa acts as a scaffold to connect the actin cytoskeleton to more than 60 functional cellular proteins, including membrane receptors (16), signaling molecules (12, 17), and DNA repair proteins (18), thus facilitating intracellular communication (9). HIV-1 entry into lymphocytes requires the activity of actin adaptors to stabilize and reorganize F-actin (19). F-actin, in turn, facilitates HIV-1 transmission from cell to cell by binding to HIV receptors and coreceptors and regulating their clustering on the cell surface (20). Our previous study found that FLNa expression is activated during the early stage of HEV infection at 4 h post-infection (hpi) but inhibited at 24 hpi (21). The knockdown of FLNa by siRNA facilitates HEV replication, indicating that FLNa is involved in HEV infection. However, the role of FLNa during the life cycle of HEV has rarely been reported.

In the present study, we evaluated the interaction between FLNa and HEV infection in patients with HEV infection, animal models, and cell cultures to explore its molecular mechanism. Notably, we found that HEV regulated the expression of FLNa negatively to inhibit the NF-κB signaling pathway to escape host antiviral responses and thus facilitate its replication. The discovery of the interaction between HEV and FLNa may provide new insights for HEV studies.

## MATERIALS AND METHODS

### Sample collection

Liver tissue sections were collected from patients between 2019 and 2025 to investigate the prevalence of HEV in Yunnan Province, China. Liver tissues from patients with hepatocellular carcinoma (HCC) and positive (*n* = 17, genotype 4) or negative (*n* = 40) for HEV RNA were employed.

SPF BALB/c mice (male, 6 weeks old, 20 g–22 g, *n* = 15) were purchased from Kunming Medical University and maintained in a pathogen-free animal facility. Mice negative for anti-HEV antibodies (IgG and IgM) and HEV RNA were included. They were divided into two groups: the uninfected control (*n* = 3) and HEV-infected (*n* = 12) groups. HEV-infected animals were necropsied at 7, 14, and 21 dpi.

### shRNA and plasmids

Two shRNAs targeting FLNa were synthesized. Their sequences were shRNA-Filamin1: 5′-GCACCGGTCCAACAAGGTCAAAGTATAACCGGTCG-3′ and shRNA-Filamin2: 5′-GCACCGGTGCAGGAGGCTGGCGAGTATACCGGTCG-3′ (20).

Plasmids expressing individual HEV ORFs, namely, pcDNA3.1-Met, pcDNA3.1-Y, pcDNA3.1-PCP, pcDNA3.1-HPR, pcDNA3.1-Hel, pcDNA3.1-RdRp, pcDNA3.1-ORF2, and pcDNA3.1-ORF3, were constructed in pcDNA3.1 vectors as described in our previous study (22).

### Cells and transfection

HepG2, HeLa, Huh7, LX2, and HEK293T cell lines were obtained from the American Type Culture Collection. Cells were maintained in Dulbecco’s modified Eagle’s medium (Servicebio, China) supplemented with 10% (vol/vol) fetal bovine serum (NEWZERUM, China), 100 U/mL penicillin, and 100 µg/mL streptomycin at 37°C under 5% CO_2_.

HepG2 cells at 70%–80% confluence were transfected with shRNAs targeting FLNa in accordance with the manufacturer’s directions to knock down FLNa. The culture medium was replaced with fresh medium at 6 h post-transfection. Cells were harvested at 72 h post-transfection, and the supernatants were subjected to RNA isolation for gene quantification. Cells were lysed with RIPA buffer for Western blot analysis.

### Viruses and viral challenge

Cell cultures were performed with genotype 1 HEV (GT1, Sar-55 strain, GenBank no. M80581.1), genotype 3 HEV (GT3, Kernow-C1/p6 strain, GenBank no. JQ679013.1), genotype 3 HEV (GT3, JDY5 strain, GenBank no. FJ527832.2), and genotype 4 HEV (GT4, KM01 strain, GenBank no. KJ155502) to investigate the interaction between HEV and FLNa. In brief, HepG2 or HeLa cells were inoculated separately with either GT1, GT3, or GT4 HEV at a multiplicity of infection = 0.1. The detailed protocol of HEV inoculation has been reported in our previous studies (23, 24). Cells were collected at indicated time points for gene quantification, indirect immunofluorescence assay (IFA), or Western blot analysis.

### Gene Ontology (GO) function, Kyoto Encyclopedia of Genes and Genomes (KEGG) pathway, and protein–protein interaction (PPI) network analyses

GO and KEGG pathway enrichment analyses were performed to characterize the functional attributes (biological process, molecular function, and cellular component) and pathway associations of differentially expressed proteins (DEPs). DEPs were identified in HepG2 cells infected with HEV for 6 days by using iTRAQ, as described in our previous paper (21). Raw mass spectrometry data were processed with Proteome Discoverer 2.1 (Thermo Fisher Scientific Inc.) and deposited in the iProx database (accession: IPX0004999000; https://www.iprox.cn). PPIs among DEPs were analyzed by employing the STRING database. The resulting interaction networks were visualized with Cytoscape software (v.3.7.1).

### HEV RNA detection and viral titer and gene quantification

Total RNA was extracted from cell cultures and liver tissues by using TRIzol Reagent (9109, Takara, Japan) in accordance with the manufacturer’s instructions. Reverse transcription PCR (RT-PCR) was performed by utilizing M-MLV reverse transcriptase with HEV-specific primers (25). HEV RNA was quantified by employing SYBR Green-based quantitative RT-PCR (qRT-PCR), as previously described (26). The expression levels of host genes associated with the NF-κB signaling pathway were quantified with gene-specific primers (Table 1). GAPDH served as the housekeeping control. Relative gene expression was calculated through the 2^−(ΔCt of gene − ΔCt of GAPDH)^ method, where Ct represents the threshold cycle. qRT-PCR was performed with the Bio-Rad CFX96 Real-Time PCR System.

**TABLE 1:** Detection of primer sequences by qRT-PCR

Name	Sequence
I-κBα-F	5′TGTCAACAGGGTAACCTACCA3′
I-κBα-R	5′ACATTCTTTTTGCCACTTTCC3′
*RIG-I*-F	5′CTGGACCCTACCTACATCCTG3′
*RIG-I*-R	5′GGCATCCAAAAAGCCACGG3′
*MDA5*-F	5′ACCAAATACAGGAGCCATGC3′
*MDA5*-R	5′CGTTCTTTGCGATTTCCTTC3′
GAPDH-F	5′AAGTGGTCGTTGAGGGCAAT3′
GAPDH-R	5′AAGTGGTCGTTGAGGGCAAT3′

### Western blot analysis and coimmunoprecipitation (Co-IP) assay

Cells were collected at indicated time points and lysed in RIPA buffer. An equivalent amount of total protein was separated through 10% sodium dodecyl sulfate-polyacrylamide gel electrophoresis and transferred onto a nitrocellulose membrane. After nonspecific blocking with 5% skimmed milk, the membrane was separately incubated with the primary antibody, anti-FLNa antibodies (BS1128, Bioworld, USA, 1:500), IκBα (9242S, Cell Signaling, UK, 1:1,000), p-IκBα (9246S, Cell Signaling, UK, 1:1,000), Flag (ab228529, Abcam, UK, 1:1,000), K63-linked ubiquitin (K63) (ab179434, Abcam, UK, 1:2,000), or GAPDH (TDY052C, TDY BIOTECH, 1:5,000) overnight at 4°C. HRP‐conjugated IgG was used as a secondary antibody (ab228529, Abcam, UK, 1:5,000). GAPDH served as the loading control. The binding signals of the antibodies were developed with Immobilon ECL Kit (9Q09US, Millipore, Germany) and visualized with ChemiDoc MP Imaging System (Bio-Rad, UK).

For the Co-IP assay, cells were lysed with RIPA lysate buffer. Whole cell lysates were incubated with the indicated antibodies at 4°C. The lysate–antibody mixture was added to protein A/G sepharose beads (Santa Cruz Biotechnology) and incubated overnight at 4°C. Beads were pelleted by centrifugation at 4°C and washed with RIPA buffer. Immunoprecipitated proteins were eluted from the beads and subjected to Western blot analysis.

### Indirect IFA and immunohistochemistry

Cells inoculated with GT1, GT3, or GT4 HEV, transfected with the pcDNA3.1-Hel-Flag plasmid, or treated with QNZ (A4217, APExBIO, USA, IC_50_ = 11 nM) were collected at indicated time points and fixed with 4% paraformaldehyde for 10 min at 37°C. Subsequently, cells were washed three times with PBS and incubated at 37°C for 60 min with one of the following primary antibodies: anti-HEV ORF2 antibody (MAB8003, Millipore, Germany, 1:250), anti-Flag antibody (TDY001, TDYbio, China, 1:200), anti-FLNa antibody (BS1128, Bioworld, USA, 1:200), or anti-β-actin (BS6007M, Bioworld, USA, 1:200). Cells were then incubated at 37°C for 45 min with secondary antibodies labeled with FITC (AS019, ABclonal, USA, 1:200) or TRITC (AS026, ABclonal, USA, 1:200). Nuclei were counterstained with DAPI (C1006, Beyotime, China) and visualized by using a laser scanning confocal microscope (Olympus SpinSR, Japan).

For immunohistochemical analysis (IHC), HEV-infected cells and liver sections from BALB/c mice or patients with HCC (3 µm) were treated with 3% H_2_O_2_ for 10 min and blocked with 5% skimmed milk for 30 min. Sections were separately incubated with anti-HEV ORF2 or anti-FLNa at 4°C overnight. After three cycles of washing with PBS, sections were incubated with HRP-conjugated secondary antibodies. The tissue sections were viewed by using a microscope (Nikon, E200, Japan).

The tyramide signal amplification (TSA)-based multiple IF (mIF) technique was performed in accordance with a cyclic staining protocol with tyramide-conjugated fluorophores by using TSA mIHC Assay Kit (G1236, Servicebio, China). In brief, HEV-infected cells and liver sections from BALB/c mice or patients with HCC (3 μm) were subjected to four sequential rounds of staining with FLNa, K63, HEV ORF2, or HNF4α (MA1-199, Thermo Fisher, USA, 1:200) followed by incubation with a secondary HRP-conjugated polymer. Signal amplification was achieved with TSA-Opal fluorophores. Between each round of staining, a heat-induced epitope retrieval step was performed to remove primary–secondary–HRP complexes before staining with the next primary antibody. After the final round of antibody staining, slides were counterstained with DAPI and mounted with ProLong Diamond antifade mounting medium. Sections were visualized by using an Olympus VS200 slide scanner (Olympus VS200, Japan).

### Terminal deoxynucleotidyl transferase dUTP nick end labeling (TUNEL) assay

Apoptotic cells in tissues were evaluated *in situ* by using a One Step TUNEL assay kit (C1086, Beyotime, China). In brief, tissue sections were deparaffinized, rehydrated, and digested with DNase and proteinase K (20 μg/mL) for 30 min at 37°C. The slides were incubated with the TUNEL reagent for 60 min at 37°C. Nuclei were counterstained with DAPI. Next, the TUNEL specimens were observed under a fluorescence microscope (Nikon Ts2, Japan).

### Macrophage (Kupffer cell) isolation

Fresh Kupffer cells were isolated from the livers of HEV-infected BALB/c mice by collagenase digestion and Percoll density gradient centrifugation. The isolated cells were washed, sequentially stained with F4/80 antibody (Abcam, ab6640, 1:100), propidium iodide (Invitrogen, P3566, 1:1,000), and Hoechst 33,342 (Invitrogen, H3570, 1:5,000) in the dark at 4°C for 30 min, followed by incubation with a FITC-conjugated secondary antibody (Abcam, ab6730, 1:200). F4/80-positive cells were then sorted by flow cytometry (FACS Aria SORP, USA), counted, and resuspended in RPMI-DMEM complete medium supplemented with 10% FBS and 1% penicillin-streptomycin.

### Transwell assay

HepG2 cells were seeded into the lower chamber of a plate, while Kupffer cells were seeded onto the membrane of a Transwell insert. The insert was then placed into the plate containing the HepG2 cells. A common culture medium was added to both compartments, and the co-culture system was incubated at 37°C with 5% CO_2_ for 48 and 72 h.

### Statistical analysis

All experiments were performed at least thrice. Data are presented as mean ± standard deviation, and statistical analysis was performed by using GraphPad Prism 9 software. Student’s *t*-test (paired *t*-test) was used to determine the significance of differences between two groups. *P* < 0.05 was considered statistically significant.

## RESULTS

### HEV inhibits FLNa expression

**Fig 1:**
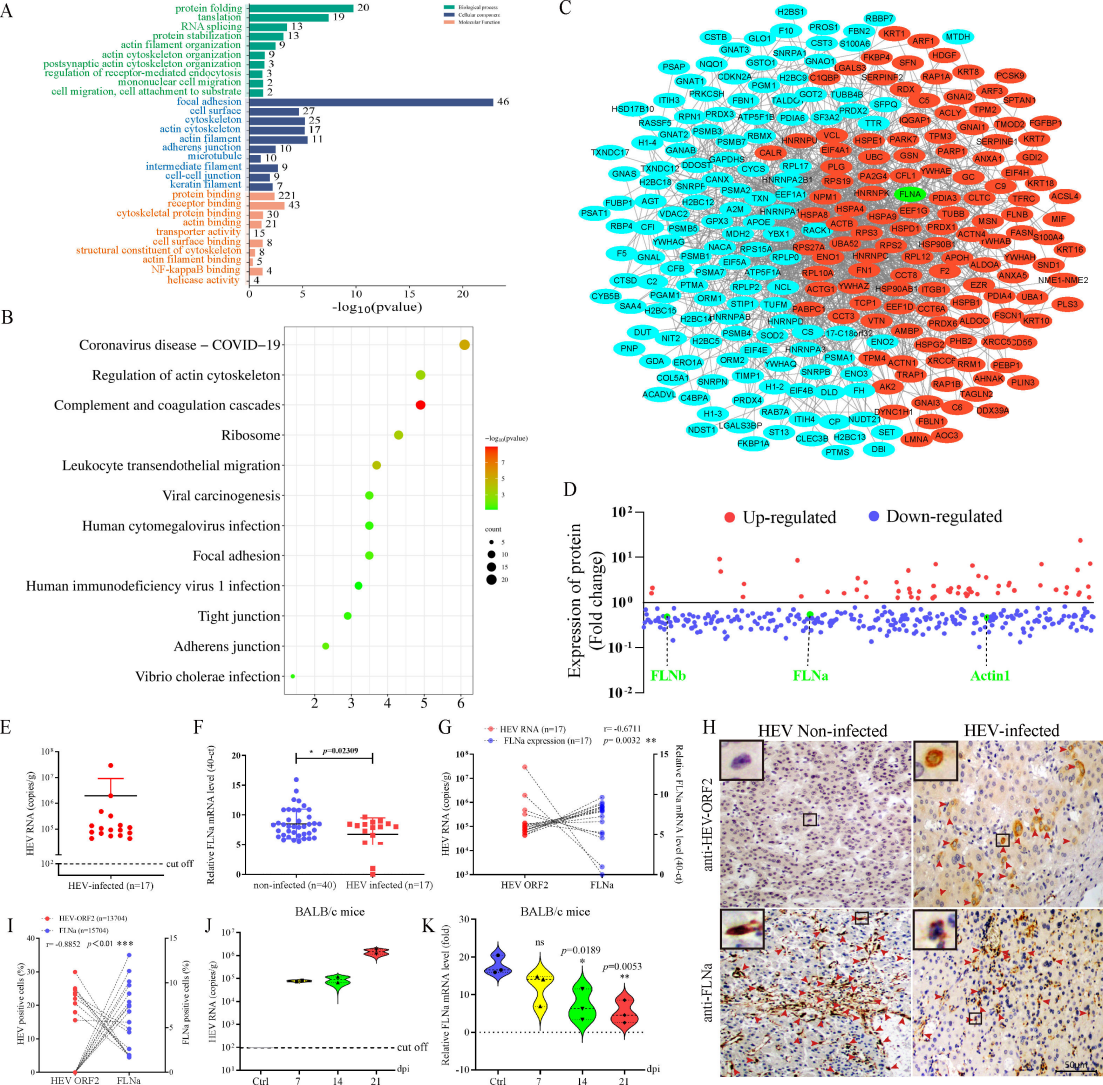
HEV infection inhibits FLNa expression *in vivo*. (**A**) Functional classification of DEPs associated with viral infection and the cytoskeleton by GO enrichment. (**B**) KEGG pathway analysis was conducted to identify the DEPs associated with HEV infection and the cytoskeleton. (**C**) DEPs in HepG2 cells infected with HEV for 6 days were detected by iTRAQ. PPI networks were built by using the STRING database and Cytoscape software. Each node in the interaction network represents a DEP. Red nodes indicate cytoskeleton DEPs involved in HEV infection. Green nodes are associated with FLNa. (**D**) Fold change of cytoskeleton-related DEPs during HEV infection. Red dots represent upregulated DEPs, blue dots represent downregulated DEPs, and green dots indicate FLNa. (**E**) HEV copy number in the livers of patients with HCC quantified by qRT-PCR. (**F**) Relative FLNa expression in the livers of patients with HCC with or without HEV infection. (**G**) Correlation between HEV copy number and FLNa expression in livers from patients with HEV infection and HCC. (**H**) IHC of HEV antigens and FLNa expression in the livers of patients with HCC with or without HEV infection. (**I**) Correlation between the percentage of HEV-positive cells (%) and FLNa-positive cells (%) in the livers of HEV-infected patients with HCC. HEV RNA copy numbers in the livers of HEV-infected BALB/c mice (**J**). Relative expression of FLNa in the livers of HEV-infected BALB/c mice (**K**). *P < 0.05; **P < 0.01; ***P < 0.001.

As described in our previous study, HEV infection inhibits FLNa expression (21). HepG2 cells infected with HEV for six days were detected by iTRAQ to identify the DEPs in mock and HEV-infected cells and to verify the effect of HEV infection on FLNa. GO enrichment analysis indicated that protein folding, translation, actin filament organization, and actin cytoskeleton organization were the most important enriched GO biological processes (Fig. 1A). Focal adhesion, cell surface, actin cytoskeleton, and actin filament were the most significantly enriched GO cellular components. Protein folding was the most significantly enriched GO molecular function (Fig. 1A). KEGG pathway analysis revealed that most of the enriched KEGG pathways focused on viral infection, actin cytoskeleton regulation, focal adhesion, and tight junctions (Fig. 1B), indicating that HEV infection affected the host cytoskeleton system. PPI networks were analyzed by using the STRING database and visualized with the NetworkX package in Python to explore the biological relevance of DEPs further. PPI network analysis revealed that 37% of the DEPs were involved in the cytoskeleton, adhesion, migration, and filaments (red dots, Fig. 1C). This finding indicated that HEV infection changed the cytoskeleton extensively. FLNa plays an important role in maintaining the tight connection of the whole cytoskeletal network. Notably, the expression levels of FLNa, FLNb, actin1, and other cytoskeleton-associated proteins were downregulated after HEV infection (Fig. 1D). These results demonstrated that HEV infection changed the cytoskeleton extensively.

The copy number of HEV and relative expression of FLNa in the liver of patients with HCC and with (*n* = 17) or without HEV infection (*n* = 40) were quantified through qRT-PCR. The correlation between HEV copy number and FLNa expression was evaluated (Fig. 1E) to investigate the effect on FLNa during HEV infection. Notably, the expression of FLNa in patients with HEV infection was significantly inhibited relative to that in noninfected patients (Fig. 1F). The expression of FLNa was negatively regulated by HEV infection (*R*^2^ = −0.6711, *P* = 0.0032, Fig. 1G). FLNa is critical for the progression of several cancers, including cervical cancer (27), pancreatic cancer (28, 29), and colorectal adenocarcinoma (30). However, the expression pattern of FLNa in patients with HEV infection and HCC is unclear. Therefore, the relationship between HEV and FLNa in patients with HEV infection and HCC was further evaluated. HEV antigens and FLNa-positive cells were separately detected by IHC (Fig. 1H). The correlation between HEV and FLNa was analyzed on the basis of the percentage of HEV- and FLNa-positive cells in the livers of patients with HCC. FLNa was consistently found to be significantly negatively regulated in patients with HEV infection and HCC (*R*^2^ = −0.8856, *P* < 0.01, Fig. 1I). The inhibition of FLNa by HEV infection was further confirmed in HEV-infected BALB/c mice at 7, 14, and 21 dpi (Fig. 1J and K). These results strongly indicated that HEV infection inhibited the expression of FLNa *in vivo*.

### HEV infection inhibits FLNa expression *in vitro*

**Fig 2:**
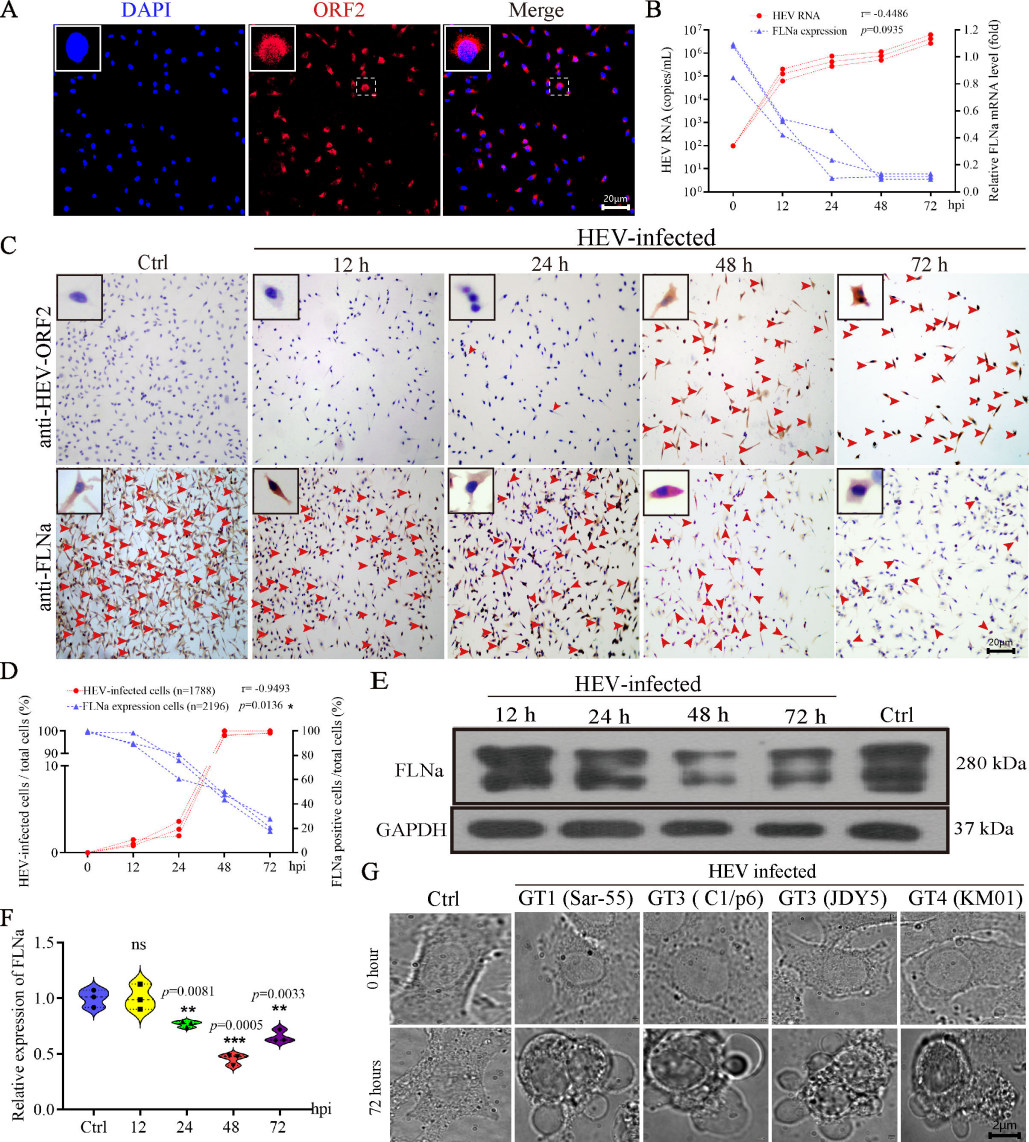
FLNa is significantly inhibited by HEV infection *in vitro*. (**A**) IFA revealing HEV replication in HepG2 cells at 48 hpi (Nikon Eclipse Ts2). HEV ORF2 (red). Nuclei stained with DAPI (blue). (**B**) Correlation between HEV copy number and relative FLNa expression in HepG2 cells at 0, 12, 24, 48, and 72 hpi. (**C**) HEV antigens and FLNa were separately detected in HepG2 cells by IHC at 12, 24, 48, and 72 hpi. (**D**) Correlation between the percentage of HEV-antigen-positive cells (%) and FLNa-positive (%) HepG2 cells. (**E**) FLNa protein expression in HEV-infected cells at 12, 24, 48, and 72 hpi determined through Western blot analysis. GAPDH served as the loading control. (**F**) Relative expression of FLNa analyzed by using ImageJ software. *P < 0.05; **P < 0.01; ***P < 0.001. (**G**) CPE in GT1 (Sar-55), GT3 (Kernow-C1/P6 and JDY5), and GT4 (KM01) HEV-infected HepG2 cells at 72 hpi observed under label-free live-cell microscopy (IDT SC3000-pro, Zircon Optics Inc).

HEV-infected HepG2 cells were used to investigate the role of FLNa in HEV replication. As revealed by IFA, HEV had successfully replicated in HepG2 cells at 72 hpi (Fig. 2A). Subsequently, HEV copy number and relative FLNa expression levels were quantified through qRT-PCR at 12, 24, 48, and 72 hpi. Consistent with that in patients with HEV infection, FLNa was significantly inhibited in HEV-infected cells from 12 hpi to 72 hpi (end of the experiment, Fig. 2B). FLNa significantly decreased upon HEV infection. However, almost no FLNa was detected at 48 hpi, when 75% of cells were infected with HEV (Fig. 2C). IHC and Western blot analysis revealed that HEV infection negatively regulates FLNa expression, with a significant inhibition observed at 24, 48, and 72 hpi (Fig. 2D through F). Since FLNa is critical for cytoskeletal integrity, its downregulation leads to a collapse of the cytoskeletal architecture. This collapse itself constitutes a direct cytopathic effect (CPE), which was captured in live-cell microscopy of cells infected with multiple HEV genotypes, including GT1 (Sar-55), GT3 (Kernow-C1/P6, JDY5), and GT4 (KM01) (Fig. 2G). Together, these results confirm that FLNa is negatively regulated by HEV infection and link this regulation to a key viral CPE.

### HEV helicase interacts with FLNa

**Fig 3:**
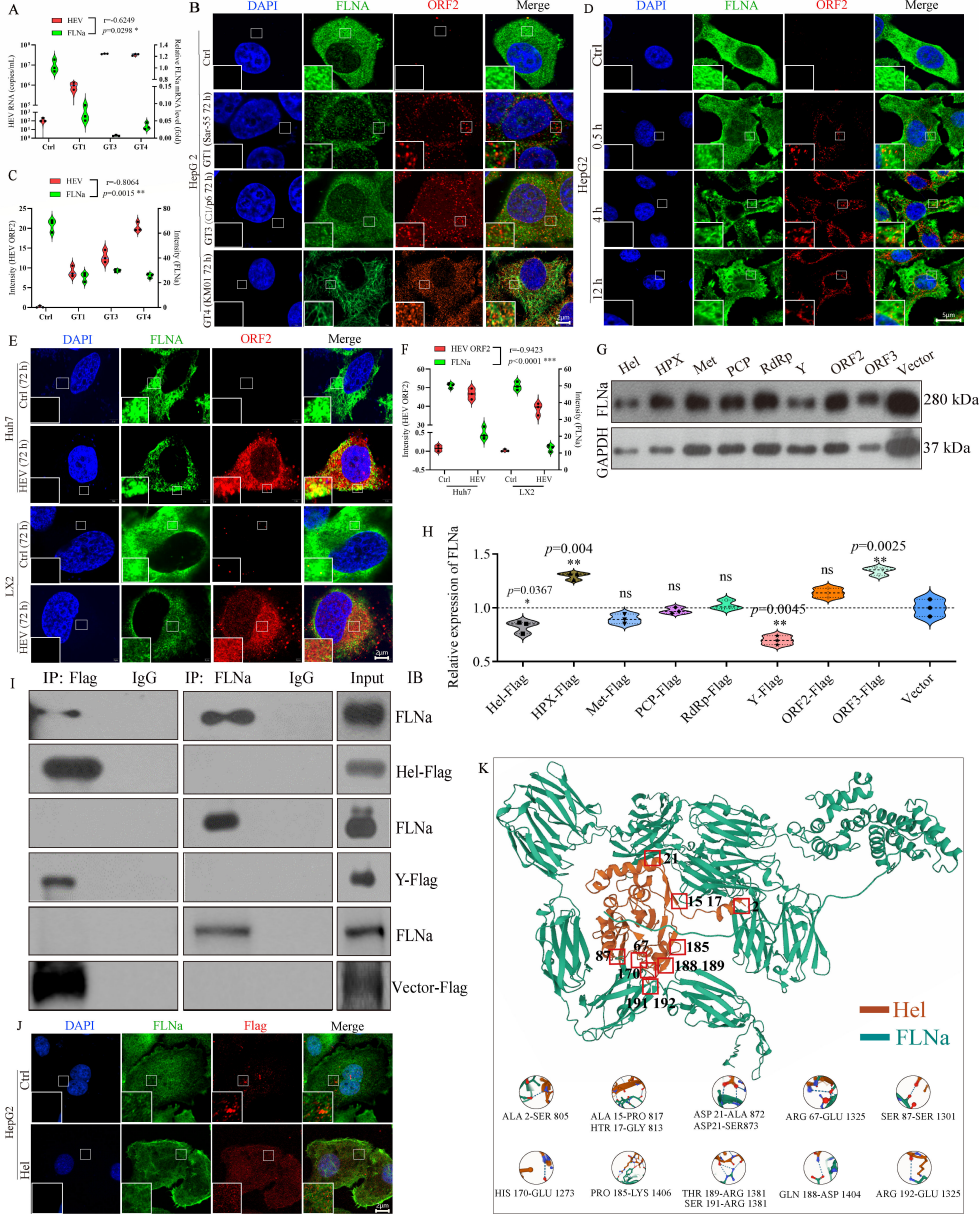
HEV helicase interacts with FLNa. (**A**) Negative correlation between HEV copy number and FLNa expression in HepG2 cells infected with GT1, GT3, or GT4 HEV at 72 hpi. (**B**) Colocalization of FLNa (green) and HEV ORF2 (GT1, GT3, or GT4 HEV, red) observed by using confocal fluorescence microscopy. (**C**) Intensity of HEV ORF2 and FLNa in HepG2 cells analyzed by ImageJ software. (**D**) Colocalization of GT4 HEV (red) and FLNa (green) observed at indicated time points by using confocal fluorescence microscopy. (**E**) Colocalization of GT4 HEV (red) and FLNa (green) in Huh7 and LX2 cells observed by using confocal fluorescence microscopy at 72 hpi. (**F**) Intensity of HEV ORF2 and FLNa in Huh7 and LX2 cells analyzed by ImageJ software. (**G**) HEK293T cells were separately cotransfected with pcDNA3.1-FLNa and pcDNA3.1-Met-Flag, pcDNA3.1-Y-Flag, pcDNA3.1-PCP-Flag, pcDNA3.1-HPX-Flag, pcDNA3.1-Hel-Flag, pcDNA3.1-RdRp-Flag, pcDNA3.1-ORF2-Flag, and pcDNA3.1-ORF3-Flag and collected at 72 hpt for Western blot analysis. GAPDH served as the loading control. (**H**) Relative expression of FLNa analyzed by ImageJ. *P < 0.05; **P < 0.01; ***P < 0.001. (**I**) Co-IP assay was performed on cells cotransfected with pcDNA3.1-FLNa and pcDNA3.1-Hel-Flag, pcDNA3.1-Y-Flag, or empty pcDNA3.1-Flag. (**J**) Colocalization of FLNa (green) and Hel (red) observed by using confocal fluorescence microscopy. (**K**) Interaction between FLNa (green) and HEV helicase (brown) predicted by AlphaFold 3.

To investigate the interaction between HEV (genotypes 1, 3, and 4) and FLNa, we analyzed the correlation between the HEV copy number and FLNa expression. A significant negative correlation was observed at 72 hpi (*r* = −0.6294, *P* = 0.0298; Fig. 3A). Furthermore, colocalization of FLNa (green) with the HEV ORF2 protein (red) in HepG2 cells infected with multiple HEV genotypes was observed (Fig. 3B). Consistent with these findings, a more pronounced negative regulation of FLNa expression by HEV infection was confirmed in a separate analysis (*r* = −0.8064, *P* = 0.0015; Fig. 3C). To investigate the HEV-FLNa interaction in detail, we performed colocalization analysis in GT4 HEV-infected HepG2 cells at 0.5, 4, and 12 hpi. Confocal microscopy revealed that the HEV ORF2 protein (red) was present in the cytoplasm within 30 min of infection and had already colocalized with FLNa (green) at this early time point (Fig. 3D). To assess the HEV-FLNa interaction across relevant cell models, both Huh7 and LX2 cell lines were utilized. The interaction was robustly associated with a significant downregulation of FLNa in both cell types (Fig. 3E and F).

Plasmids encoding each HEV domain (pcDNA3.1-Met-Flag, pcDNA3.1-Y-Flag, pcDNA3.1-PCP-Flag, pcDNA3.1-HPX-Flag, pcDNA3.1-Hel-Flag, pcDNA3.1-RdRp-Flag, pcDNA3.1-ORF2-Flag, and pcDNA3.1-ORF3-Flag) were separately cotransfected along with pcDNA3.1-FLNa into HEK293T cells for 72 h to identify the HEV domain that interacts with FLNa. Western blot analysis showed that FLNa was significantly inhibited under transfection with the plasmids encoding Hel and Y (Fig. 3G and H). Subsequently, Co-IP was performed on cells cotransfected with FLNa and Hel, and with FLNa and Y. Proteins isolated from cells cotransfected with pcDNA-3.1-FLNa at 72 post-transfection were pulled down by using an anti-Flag antibody and subsequently detected by using the FLNa antibody (Fig. 3I). No signal was observed in cells transfected with pcDNA3.1-Y-Flag (or empty vector) and FLNa. The Co-IP results illustrated that HEV helicase domain serves as the primary binding partner for FLNa. Colocalization between HEV helicase and FLNa further confirmed this interaction (Fig. 3J). Furthermore, the detailed interaction sites of FLNa and HEV helicase were predicted by using AlphaFold 3 (https://golgi.sandbox.google.com/, Fig. 3K). Twelve potential interaction sites on HEV helicase (residues 2, 15, 17, 21, 67, 87, 170, 185, 188, 189, 191, and 192) were predicted to interact with FLNa.

### Knockdown of FLNa facilitates HEV replication

**Fig 4:**
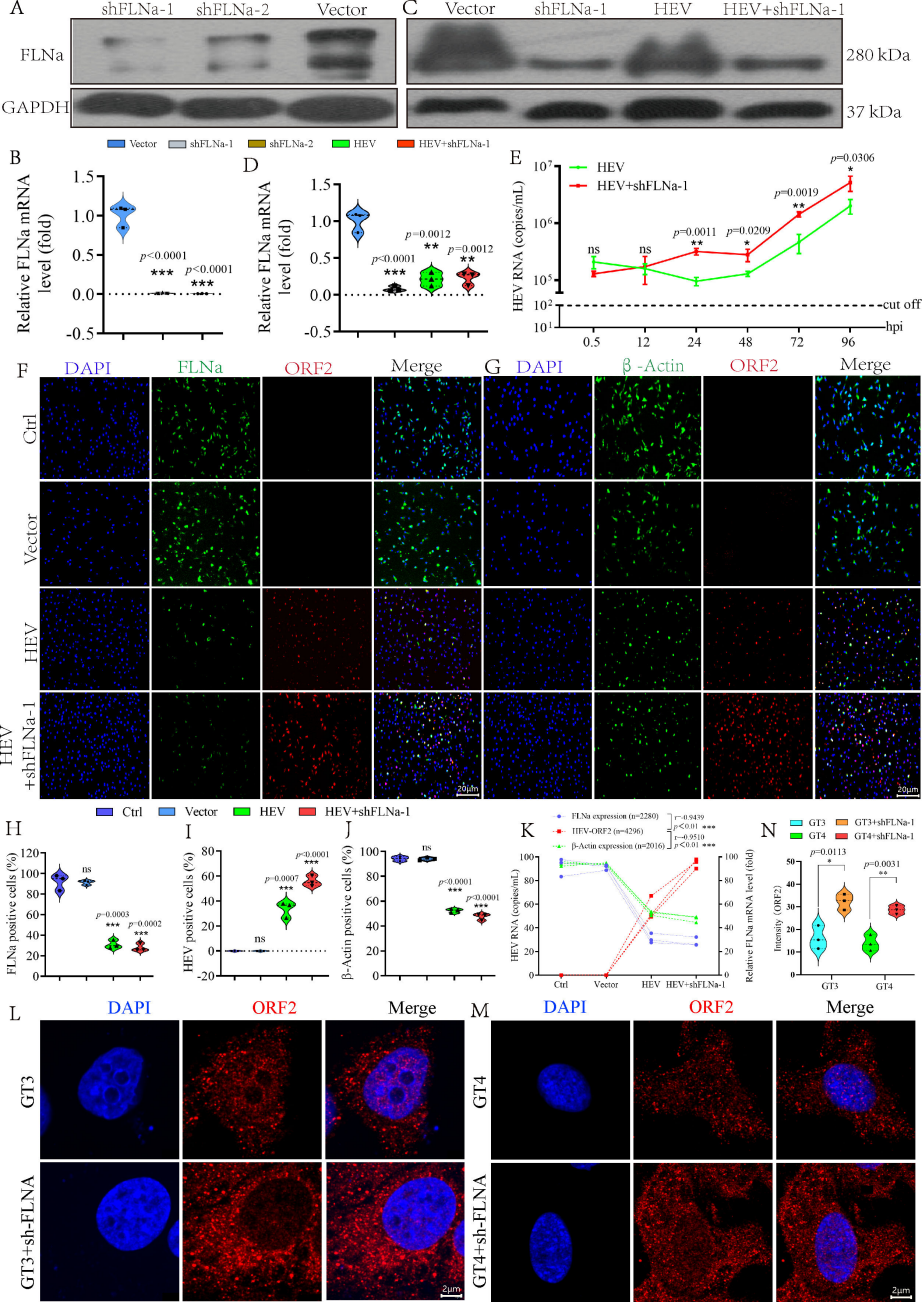
Knockdown of FLNa facilitates HEV replication. (**A**) FLNa expression in cells transfected with shFLNa-1, shFLNa-2, or the vector for 72 h determined by Western blot analysis. GAPDH served as the loading control. (**B**) Fold change of FLNa in HepG2 cells transfected with shFLNa-1, shFLNa-2, or the vector at 72 hpi quantified by qRT-PCR. (**C**) FLNa expression in HEV-infected cells with or without shFLNa-1 pretransfection detected by Western blot analysis. GAPDH served as the loading control. (**D**) Fold change of FLNa in HEV-infected HepG2 cells with or without shFLNa-1 pretransfection at 24 h before infection. (**E**) qRT-PCR detection of the copy number of HEV in HepG2 cells pretransfected with shFLNa-1 for 24 h before HEV infection. (**F**) IFA results showing that the knockdown of FLNa (green) facilitated HEV replication (ORF2, red). (**G**) IFA results indicating that the knockdown of FLNa decreased β-actin expression (green) and facilitated HEV replication (red). Percentages of FLNa-positive cells (**H**), HEV-positive cells (**I**), and β-actin-positive cells (**J**). (**K**) Correlation among HEV, FLNa, and β-actin in HEV-infected cells. Expression levels of HEV ORF2 antigens in HepG2 cells inoculated with GT3 HEV (**L**) or GT4 HEV (**M**) observed by using confocal fluorescence microscopy. (**N**) Intensity of HEV ORF2 in cells inoculated with GT3 HEV or GT4 HEV analyzed by ImageJ software. *P < 0.05; **P < 0.01; ***P < 0.001.

FLNa was knocked down by using RNAi technology to assess the effect of its inhibition on HEV replication. shRNAs (shFLNa-1 and shFLNa-2) targeting FLNa were separately transfected into HepG2 cells for 72 h. Western blot analysis (Fig. 4A) and qRT-PCR (Fig. 4B) identified the significant silencing of FLNa. Given that shFLNa-1 significantly inhibited FLNa at the mRNA and protein levels, it was employed to perform the subsequent knockdown experiments. The inhibition of FLNa at the mRNA (Fig. 4D) and protein (Fig. 4C) levels was aggravated in cells pretransfected with shFLNa-1 then infected with HEV. Notably, at 24, 48, 72, and 96 hpi, the viral titers in cells with FLNa knockdown through shFLNa-1 were significantly higher than those in cells infected with HEV alone (Fig. 4E). The promotion of HEV replication by FLNa knockdown was further identified by IFA. Remarkably, FLNa expression (green) was significantly suppressed by HEV infection and frustrated under pretransfection with shFLNa-1 followed by HEV infection at 72 hpi (Fig. 4F and H). HEV ORF2 antigens (red) in cells with shFLNa-1-induced FLNa knockdown had significantly increased relative to those in the control cells without transfection or cells transfected with the vector (Fig. 4F and I). β-Actin (green), another critical cytoskeletal protein that cross-links with FLNa via its N-terminal actin-binding domain, also decreased at 72 h of HEV infection (Fig. 4G and J). Correlation analysis further confirmed that HEV replication negatively regulated the expression levels of FLNa (*R* = −0.9439, *P* < 0.01, Fig. 4K) and β-actin (*R* = −0.9510, *P* < 0.01, Fig. 4K), indicating that HEV infection perturbed the host cytoskeleton. Furthermore, IFA confirmed that GT3 and GT4 HEV replication significantly increased in HepG2 cells with FLNa knockdown (Fig. 4L through N). These results revealed that HEV disturbed the host cytoskeletal network to facilitate its replication.

### HEV infection inhibited FLNa expression to escape from recognition by the NF-κB signaling pathway

**Fig 5:**
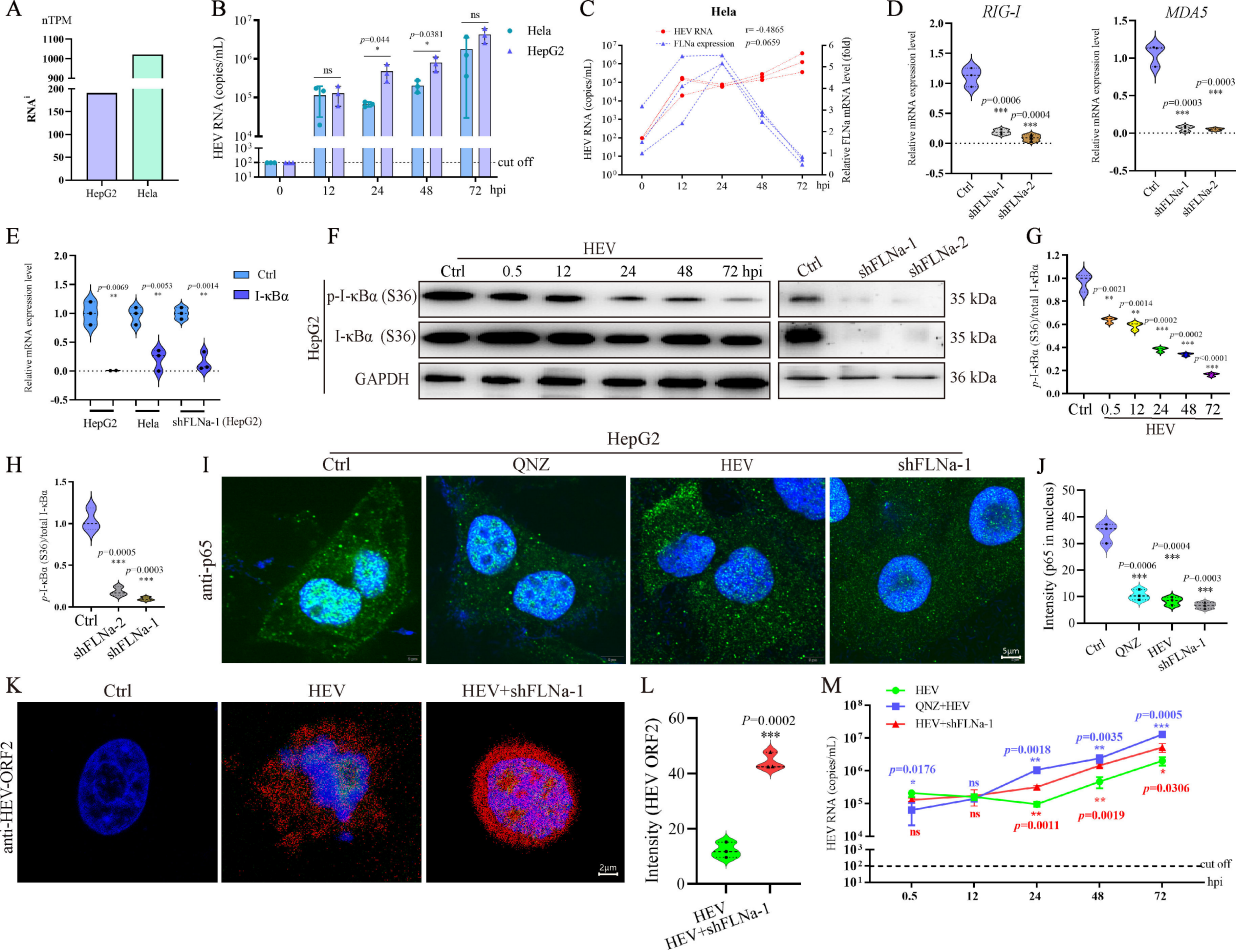
HEV infection inhibits FLNa expression to escape recognition by the NF-κB signaling pathway. (**A**) Expression of FLNa in HepG2 and HeLa cells. (**B**) Correlation between HEV copy number and FLNa expression in HeLa cells at indicated time points. (**C**) HEV copy number in HeLa and HepG2 cell lines at indicated time points. (**D**) mRNA expression levels of RIG-I and MDA5 in HepG2 cells with FLNa knockdown by shRNA. (**E**) mRNA expression of I-κBα in HepG2 cells, HeLa cells, or HepG2 cells with FLNa knockdown quantified by qRT-PCR. (**F**) Total I-κBα and *p-*I-κBα levels in HEV-infected cells or FLNa knockdown cells (shFLNa) detected by Western blot analysis at indicated time points. GAPDH served as the loading control. Relative expression level of *p*-I-κB/I-κB normalized to total I-κBα in HEV-infected HepG2 cells (**G**) and in cells with FLNa knockdown (**H**). (**I**) P65 observed in the nuclei of cells treated with QNZ, infected with HEV, or subjected to FLNa knockdown. (**J**) Intensity of p65 in nuclei analyzed by ImageJ software. (**K**) HEV antigens in HEV-infected cells with or without shFLNa-1 transfection. (**L**) Intensity of HEV antigens analyzed by ImageJ software. (**M**) HEV copy number in HEV-infected cells treated with QNZ or FLNa knockdown. *P < 0.05; **P < 0.01; ***P < 0.001.

HepG2 and HeLa cell lines with different FLNa expression levels were included to investigate the interaction between HEV infection and FLNa expression further (Fig. 5A). Furthermore, the replication efficiencies of HEV in HepG2 and HeLa cell lines with different FLNa expression levels were compared. Consistently, high HEV replication was found in HepG2 cells with low FLNa expression (Fig. 5B). A negative correlation was also observed between HEV copy number and FLNa expression in HEV-infected HeLa cells (Fig. 5C). These results further demonstrated that the deficiency of FLNa expression facilitated HEV infection.

The physical changes in cell membranes and the cytoskeleton caused by viral infection are sensed by the host to activate an innate immune response, such as the rapid and decisive NF-κB signaling pathway. Although the NF-κB signaling pathway plays a critical role during HEV infection, how the host senses HEV infection is unclear. Notably, the knockdown of FLNa significantly inhibited RIG-I and MDA5 mRNA expression (Fig. 5D). The key gene of *Nfkb* (IκBα) in the NF-κB signaling pathway in HEV-infected HepG2 and HeLa cell lines at 72 hpi was quantified by qRT-PCR to investigate the effect of FLNa on the NF-κB signaling pathway during HEV infection. HEV infection significantly inhibited IκBα expression, with its inhibitory effect being comparable to that of FLNa-targeting shRNA (Fig. 5E).

NF-κB is maintained in an inactive state within the cytoplasm through its interaction with the inhibitory proteins IκBs. The proteolytic degradation of IκBs immediately proceeds and is required for the nuclear translocation of NF-κB. HEV infection significantly suppressed the phosphorylation of I-κBα at serine 36 (*p*-I-κBα), whereas FLNa knockdown completely inhibited the expression of I-κBα and *p*-I-κBα (Fig. 5F through H). The suppression of I-κBα phosphorylation blocked the transcription of NF-κB for nuclear translocation. Consequently, IFA revealed that P65 in the nuclei of HEV-infected cells was significantly reduced to levels comparable to those in cells treated with QNZ (a small-molecule inhibitor that strongly inhibits the transcriptional activation of NF-κB) or subjected to FLNa knockdown (Fig. 5I and J). Furthermore, the knockdown of FLNa facilitated HEV replication, as further confirmed by IFA (Fig. 5K and L). The inhibition of NF-κB nuclear translocation by QNZ treatment or FLNa knockdown significantly aggravated HEV replication (Fig. 5M), further confirming that HEV escapes recognition by the NF-κB signaling pathway to facilitate viral replication. These results demonstrated that HEV-induced FLNa inhibition impaired RIG-*I* sensing in collaboration with NF-κB signaling pathway recognition. This phenomenon might be responsible for the promotion of viral replication.

### HEV inhibits FLNa-aggravated cell apoptosis through ubiquitination

**Fig 6:**
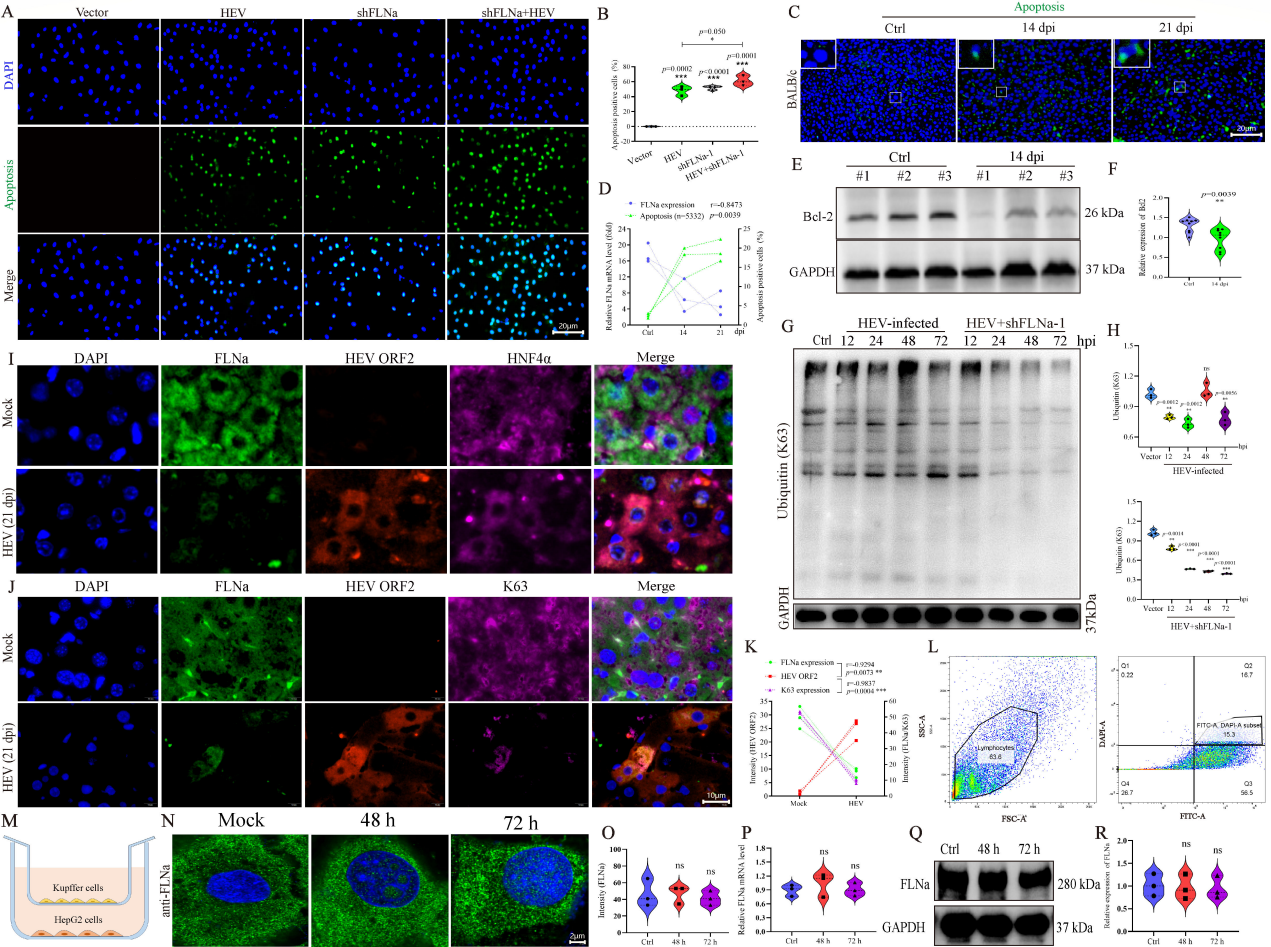
HEV inhibits FLNa-aggravated cell apoptosis through ubiquitination. (**A**) TUNEL assay performed on HEV-infected cells with or without shFLNa-1 transfection. (**B**) Percentage of apoptotic cells in HEV-infected cells with or without shFLNa-1 transfection. (**C**) TUNEL assay performed on the livers of HEV-infected BALB/c mice at 14 and 21 dpi. (**D**) Correlation between the percentage of apoptotic cells (%) and FLNa mRNA expression in the livers of HEV-infected BALB/c mice at 14 and 21 dpi. (**E**) Bcl-2 protein expression in BALB/c mice infected with HEV at 14 dpi detected by Western blot analysis. GAPDH served as the loading control. (**F**) Relative expression of Bcl-2 normalized to GAPDH and analyzed by using ImageJ software. (**G**) K63-mediated ubiquitination determined in HEV-infected cells with or without shFLNa-1 pretransfection. (**H**) Relative expression of K63 normalized to GAPDH and analyzed by using ImageJ software. (**I, J**) Expression of FLNa (green), HEV antigens (red), HNF4α (purple, Hepatocyte Nuclear Factor), and K63-mediated ubiquitination (purple) in the livers of BALB/c mice infected with HEV for 21 days detected by IF. (**K**) Intensity of HEV ORF2, FLNa, and K63 analyzed by ImageJ software. (**L**) Flow cytometry was used to isolate Kupffer cells. (**M**) Transwell assay schematic diagram. (**N**) The expression of FLNa was detected by immunofluorescence after co-culturing HepG2 cells with Kupffer cells for 48 h and 72 h. (**O**) Intensity of FLNa analyzed by ImageJ software. (**P**) The mRNA expression of FLNa after co-culturing HepG2 cells with Kupffer cells for 48 h and 72 h. (**Q**) FLNa protein expression in HepG2 cells with co-culturing by Western blot analysis. (**R**) Relative expression of FLNa normalized to GAPDH and analyzed by using ImageJ software. *P < 0.05; **P < 0.01; ***P < 0.001.

HEV infection is associated with apoptosis (31, 32). Whether HEV infection inhibits FLNa and disturbs the cytoskeletal network to affect apoptosis is unclear. Therefore, a TUNEL assay was performed on cells infected with HEV or cells with FLNa knockdown. Notably, HEV infection induced moderate apoptosis, and comparable induction was observed in cells with FLNa knockdown (Fig. 6A). Remarkably, aggravated apoptotic bodies were observed in cells pretransfected with shFLNa-1 and then infected with HEV (Fig. 6A and B). Consistently, exacerbated apoptosis was observed in the livers of HEV-infected BALB/c mice (Fig. 6C). The occurrence of apoptosis was negatively correlated with the expression of FLNa (Fig. 6D). In addition, the impairment of BCL-2, an antiapoptotic factor, was significantly inhibited in HEV-infected livers at 14 dpi (Fig. 6E and F). These findings indicated that HEV infection-induced FLNa suppression aggravated apoptosis.

Furthermore, K63-mediated ubiquitination was employed to evaluate cells infected with HEV or subjected to FLNa knockdown. Notably, HEV infection decreased ubiquitination, whereas FLNa knockdown aggravated ubiquitination degradation (Fig. 6G and H). The negative regulatory relationship between HEV and FLNa was further confirmed in liver sections from HCC patients, with HNF4α (a key hepatocyte nuclear factor) used as a liver-specific marker to identify parenchymal cells (Fig. 6I). mIF also revealed marked degradation of K63-mediated ubiquitination (purple) in HEV-infected liver sections (HEV ORF2 protein, red) along with decreased FLNa (green) (Fig. 6J and K), further confirming that HEV infection inhibited FLNa and K63-mediated ubiquitination.

To investigate whether the HEV-FLNa interaction is mediated by a paracrine mechanism, a co-culture system was established. Primary macrophages (Kupffer cells) were isolated from the livers of mice infected with HEV for 14 days by FACS (Fig. 6L). These macrophages were then co-cultured with HepG2 cells using a transwell system that permits the exchange of soluble factors but prevents direct cell contact (Fig. 6M). No significant change in FLNa protein or mRNA expression was detected in the HepG2 cells co-cultured with macrophages from HEV-infected livers, relative to those co-cultured with macrophages from mock-infected livers, at 48 and 72 h (Fig. 6N through R).

## DISCUSSION

HEV is the most common viral hepatitis virus in the world, causing acute self-limited disease in the immunocompetent general population (33) and chronic infection in immunocompromised patients (34), such as recipients of organ transplants (35), individuals infected with HIV (36), or patients being treated with immunosuppressive drugs (37). Pregnant women, especially those in their third trimester of pregnancy, are highly sensitive to HEV infection because of their immune-tolerant state, rapidly rising estrogen and progestin levels (23, 24), and increased positive regulation of binding and structural molecule activities (21). The virus binds to host receptors, which are linked to intercellular junctional proteins and the intracellular cytoskeletal network (38–40). FLNa is involved in cytoskeleton remodeling to construct a barrier to infection. It interacts with HIV virions and likely participates in viral assembly and budding (19). Although HEV infection was considered to lack a CPE, certain strains, like 87A and 93G variants, have been shown to induce marked CPE in A549 cells (41, 42). Additionally, avian HEV infection has also been linked to cytoskeleton rearrangement (43). In the present study, the substantial inhibition of FLNa during HEV infection disrupted the stabilization of the cytoskeletal network. This effect may be responsible for facilitating viral entry.

Although HEV has been isolated for more than 30 years, the mechanisms underlying its entry into host cells and sensing by pattern recognition receptors (PRRs) are largely unexplored. The virus binds to receptor(s), invades the host membrane, and is recognized by PRRs to initiate antiviral responses. NF-κB plays a key role in sensing pathogen invasion to initiate the innate immune response against viral infection (44). However, the disruption of FLNa inhibits the proteolytic degradation of IκB, blocking the nuclear translocation of NF-κB. As a result, attenuated or no innate immune responses are induced to clear the virus. Moreover, robust viral replication occurs, and numerous virions are released. The remarkable differential expression of FLNa was first found in a study that used proteomic analysis to clarify the mechanism underlying the increased sensitivity of pregnant women to HEV infection. The disrupted cytoskeletal barrier to the virus, the blinding of innate immunity based on NF-κB, the facilitation of viral replication by estrogen and progestin, and immune tolerance accelerate HEV infection and replication.

In general, viruses alter host signaling pathways to create a suitable environment for their infection and replication. For example, HEV ORF1, which encodes PCP, has been reported to inhibit IFN-I activation by suppressing the K63-linked deubiquitination of retinoic acid-inducible gene I and TANK-binding kinase 1 (45). HEV helicase is involved in unwinding double-stranded RNA, RNA processing (46), transcriptional regulation, and pre-mRNA processing and initiates the first step of 5′ cap synthesis (47). In the present study, we found that the HEV helicase domain as the primary binding partner for FLNA provides mechanistic insight and will guide our future studies with the full-length protein. The interaction between HEV and FLNa disturbs the cytoskeleton barrier and blocks the activation of the NF-κB signaling pathway and thus facilitates HEV replication. Additional attention should be paid to clarify the detailed interactions between HEV and the cytoskeleton to explore the entry and replication of HEV. Such an exploration will benefit the understanding of the HEV life cycle and exploration of novel therapeutic strategies.

In conclusion, HEV significantly inhibited FLNa expression *in vivo* and *in vitro* to escape sensing by the RIG-I and NF-κB signaling pathways, thus facilitating HEV replication. Furthermore, the knockdown of FLNa diminished ubiquitination to aggravate apoptosis and inflammatory responses.

## Data Availability

The data that support the findings of this study are available from the corresponding author upon reasonable request.
